# Development and validation of a nomogram for predicting in-hospital death in cirrhotic patients with acute kidney injury

**DOI:** 10.1186/s12882-024-03609-8

**Published:** 2024-05-21

**Authors:** Xiang Li, Xunliang Li, Wenman Zhao, Deguang Wang

**Affiliations:** 1 Department of Nephrology, The Second Affiliated Hospital of Anhui Medical University, Hefei, China; 2 Department of Nephrology, Affiliated Hospital of Jining Medical University, Jining, China

**Keywords:** Acute kidney injury, Cirrhosis, Nomogram, Prognosis, Mortality

## Abstract

**Background:**

The purpose of this study was to develop a nomogram for predicting in-hospital mortality in cirrhotic patients with acute kidney injury (AKI) in order to identify patients with a high risk of in-hospital death early.

**Methods:**

This study collected data on cirrhotic patients with AKI from 2008 to 2019 using the Medical Information Mart for Intensive Care IV. Multivariate logistic regression was used to identify confounding factors related to in-hospital mortality, which were then integrated into the nomogram. The concordance index (C-Index) was used to evaluate the accuracy of the model predictions. The area under the curve (AUC) and decision curve analysis (DCA) was used to assess the predictive performance and clinical utility of the nomogram.

**Results:**

The final study population included 886 cirrhotic patients with AKI, and 264 (29.8%) died in the hospital. After multivariate logistic regression, age, gender, cerebrovascular disease, heart rate, respiration rate, temperature, oxygen saturation, hemoglobin, blood urea nitrogen, serum creatinine, international normalized ratio, bilirubin, urine volume, and sequential organ failure assessment score were predictive factors of in-hospital mortality. In addition, the nomogram showed good accuracy in estimating the in-hospital mortality of patients. The calibration plots showed the best agreement with the actual presence of in-hospital mortality in patients. In addition, the AUC and DCA curves showed that the nomogram has good prediction accuracy and clinical value.

**Conclusions:**

We have created a prognostic nomogram for predicting in-hospital death in cirrhotic patients with AKI, which may facilitate timely intervention to improve prognosis in these patients.

## Introduction

Acute kidney injury (AKI) is a common and severe complication of cirrhosis, characterized by a rise in serum creatinine (Scr) levels or a decrease in urine output [1, 2]. In some investigations, the incidence of AKI in patients with cirrhosis was between 20% and 50% [2, 3]. In addition, some studies have recorded mortality rates as high as 80% for cirrhotic patients with AKI [4, 5]. Therefore, identifying patients with cirrhosis and AKI at a higher risk of death is essential and may help improve the prognosis of these patients through the timely implementation of medicinal therapies.

Nomograms are straightforward statistical visualization tools that can estimate the likelihood of a particular result. Nomograms are currently utilized extensively for the diagnosis of diseases and the estimation of mortality [6–9]. However, nomograms have been utilized seldom to predict in-hospital mortality in cirrhotic patients with AKI. Consequently, this research aimed to evaluate the risk factors for in-hospital death in cirrhotic patients with AKI and to develop a nomogram to predict the risk of in-hospital mortality in these patients, with the ultimate goal of giving possible therapeutic guidance for early diagnosis and care in high-risk patients.

## Methods

### Database introduction

The Medical Information Mart for Intensive Care IV (MIMIC IV) database is an extensive, anonymous, publicly available clinical database authorized by the Massachusetts Institute of Technology [10]. The database records clinical data on patients in the intensive care unit (ICU) at Beth Israel Deaconess Medical Center between 2008 and 2019. Individual patient consent and ethically informed consent declarations are unnecessary because the database contains no identifying information about the patients and has no bearing on clinical decision-making [11]. In order to apply for access to the database, we were granted eligibility after passing the needed assessment.

### Study population

This study included all cirrhotic patients with AKI in the ICU. The International Classification of Diseases, Ninth Revision (ICD-9) codes 5712, 5715, and 5716 were used to identify people with cirrhosis. AKI was diagnosed according to the Kidney Disease: Improving Global Outcomes (KDIGO) 2012 criteria within 48 h of ICU admission [12]: (1) Scr increased to more than 1.5 times the baseline value within the previous 7 days; (2) past 48 h Scr increased ≥ 0.3 mg/dl; or (3) urine output < 0.5 ml/kg/h for 6 h or more. The lowest Scr result within the preceding seven days was utilized as the baseline Scr level [13]. If baseline Scr values were unavailable before admission, the first Scr value recorded after admission was utilized [14]. Only the initial admission was evaluated in analyzing patients with multiple ICU admissions. Patients under the age of 18 and those with ICU stays of less than 48 h were excluded.

### Data collection

We extracted patient information from the MIMIC IV database, including etiology of cirrhosis, age, gender, weight, comorbidities, vital signs, laboratory indicators, urine output, mechanical ventilation, renal replacement therapy (RRT), vasopressors use, disease severity score and mortality. Comorbidities included congestive heart failure, peptic ulcer disease, myocardial infarction, peripheral vascular disease, diabetes, chronic pulmonary disease, rheumatic disease, cerebrovascular disease, chronic kidney disease, cancer, paraplegia, and acquired immune deficiency syndrome, ascites, and portal encephalopathy. The employed vital signs included heart rate, mean arterial pressure (MAP), respiratory rate, temperature, and oxygen saturation (SpO_2_), with the mean values from the initial 24 h after ICU admission used. For laboratory indicators, the maximum values during the first 24 h of ICU admission were used and included hematocrit, hemoglobin, platelets, white blood cell (WBC), blood urea nitrogen (BUN), anion gap, international normalized ratio (INR), Scr, serum glucose, serum calcium, serum chloride, bicarbonate, serum potassium, serum sodium, partial thromboplastin time (PTT), and prothrombin time (PT), albumin, and bilirubin. The disease severity score included sequential organ failure assessment (SOFA) score and simplified acute physiology score II (SAPS II). In addition, we used the total urine output of the patient during the first 24 h after admission to the ICU.

### Statistical analysis

In the MIMIC IV database, missing data are widespread, and to minimize severe bias, less than 20% of variables were missing in this analysis. In this work, various interpolations using the ‘mice’ package of the R software were utilized to fill in missing data.

Due to their non-normal distribution, continuous variables in this study were reported as the median and interquartile range (IQR), and the Mann-Whitney test was employed to evaluate differences between groups. Categorical variables were reported as numbers and percentages, and the chi-square test or Fisher’s exact test was used, as appropriate, to compare groups. In the multivariate logistic analysis, we considered both statistically significant factors in the univariate analysis (*p* < 0.05) and those that had previously been shown to have clinical relevance. The final logistic regression model was chosen by using backward stepwise regression. The collinearity between the final model variables was evaluated using the variance inflation factor (VIF), with VIF ≤ 5 indicating the absence of collinearity. Based on the findings of multivariate logistic regression on the dependent variable, a nomogram was generated using the ‘rms’ package of the R software. Consequently, odds ratios (OR) with 95% confidence intervals (95% CI) were calculated. The concordance index (C-Index) was used to evaluate the discriminatory ability of the model and decrease overfitting bias. We also used the area under curve (AUC) and decision curve analysis (DCA) to assess the predictive performance and clinical utility of the model. R software (version 4.2.1) was used to conduct statistical analyses. P-values < 0.05 were considered statistically significant.

## Results

### Mortality and characteristics of cirrhotic patients with acute kidney injury

**Fig. 1:**
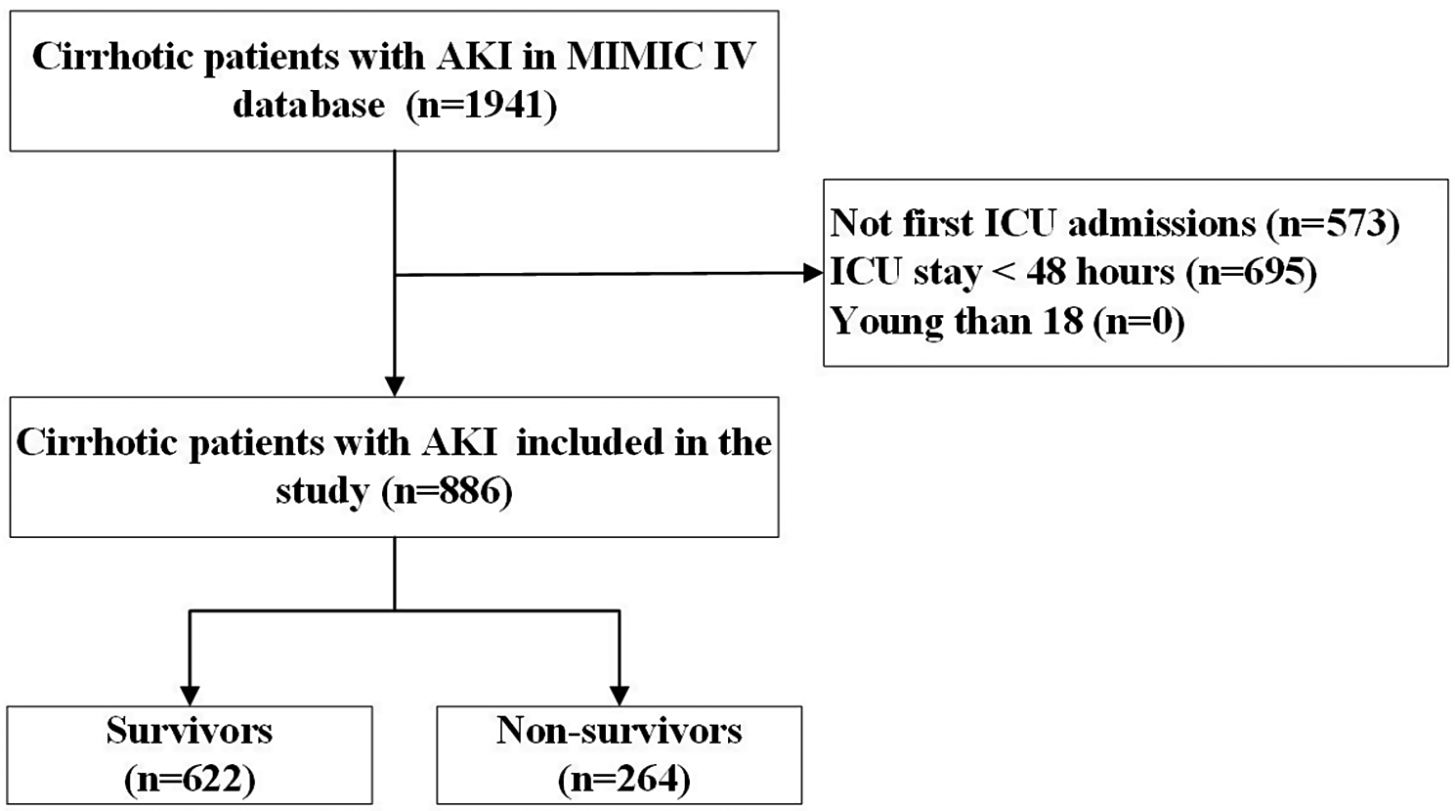
Study flowchart. *Abbreviations* MIMIC IV: Medical Information Mart for Intensive Care IV, ICU: intensive care unit, AKI: acute kidney injury

A total of 1911 cirrhotic patients with AKI who were eligible to participate were identified. 573 patients were eliminated because of multiple ICU admissions, and 695 were excluded due to ICU stays of less than 48 h. The final study population included 886 cirrhotic patients with AKI, and 264 (29.8%) died in the hospital (Fig. 1).

The incidence of cerebrovascular disease and sepsis was significantly higher in patients in the non-survivor group versus those in the survivor group (*p* < 0.05). In addition, the non-survivor group had a higher heart rate, respiratory rate, vasopressors use, SOFA score, and SAPS II and a lower MAP, body temperature, SpO_2_, and urine output than the survivor group (*p* < 0.05). For laboratory examinations, such as hemoglobin, platelets, WBC, anion gap, bicarbonate, BUN, serum calcium, serum chloride, Scr, INR, PT, and PTT were significantly different between the two groups (*P* < 0.05). The essential aspects of the data collection are summarized in Table 1.

**Table 1:** Baseline characteristics of survival and non-survivor in cirrhotic patients with acute kidney injury

Variables	Total (*n* = 886)	Survivors (*n* = 622)	Non-survivors (*n* = 264)	*P* value
Cause				0.401
Cholestasis or alcoholic	449 (50.7)	309 (49.7)	140 (53.0)	
Other	437 (49.3)	313 (50.3)	124 (47.0)	
Sex, male	570 (64.3)	392 (63.0)	178 (67.4)	0.240
Age (years)	59.7 [53.0, 68.0]	59.7 [52.9, 67.8]	59.6 [53.3, 68.8]	0.766
Weight (kg)	84.1 [71.6, 100.0]	83.0 [71.3, 100.0]	85.9 [72.0, 100.7]	0.292
Myocardial infarction	61 (6.9)	38 (6.1)	23 (8.7)	0.210
Congestive heart failure	169 (19.1)	119 (19.1)	50 (18.9)	1
Peripheral vascular disease	63 (7.1)	42 (6.8)	21 (8.0)	0.621
Cerebrovascular disease	69 (7.8)	34 (5.5)	35 (13.3)	< 0.001
Chronic pulmonary disease	246 (27.8)	182 (29.3)	64 (24.2)	0.149
Rheumatic disease	20 (2.3)	17 (2.7)	3 (1.1)	0.224
Peptic ulcer disease	66 (7.4)	49 (7.9)	17 (6.4)	0.545
Diabetes	260 (29.3)	182 (29.3)	78 (29.5)	0.996
Paraplegia	16 (1.8)	10 (1.6)	6 (2.3)	0.686
Chronic kidney disease	219 (24.7)	150 (24.1)	69 (26.1)	0.581
Cancer	159 (17.9)	107 (17.2)	52 (19.7)	0.430
Aids	21 (2.4)	16 (2.6)	5 (1.9)	0.715
Sepsis	513 (57.9)	340 (54.7)	173 (65.5)	0.003
Ascites	433 (48.9)	306 (49.2)	127 (48.1)	0.823
Portal encephalopathy	369 (41.6)	262 (42.1)	107 (40.5)	0.715
Heart rate (beats/minute)	86.9 [76.1, 99.5]	85.7 [75.7, 97.7]	91.7 [77.2, 103.7]	0.001
MAP (mmHg)	71.9 [66.5, 80.1]	73.0 [66.8, 81.1]	70.7 [65.5, 76.9]	< 0.001
Respiratory rate (beats/minute)	18.5 [16.1, 21.6]	17.9 [15.8, 20.8]	19.9 [17.0, 23.6]	< 0.001
Body temperature (°C)	36.7 [36.4, 37.0]	36.8 [36.5, 37.1]	36.6 [36.2, 36.9]	< 0.001
SpO_2_ (%)	97.4 [95.9, 98.8]	97.6 [96.0, 99.0]	96.9 [95.5, 98.4]	< 0.001
Hematocrit (%)	31.5 [28.2, 35.5]	31.8 [28.7, 35.5]	31.0 [27.3, 35.5]	0.064
Hemoglobin (g/dL)	10.5 [9.3, 11.8]	10.6 [9.5, 11.9]	10.3 [9.0, 11.8]	0.013
Platelets (K/uL)	123 [84, 175]	129 [89, 178]	113 [74, 165]	0.001
WBC (K/uL)	11.5 [8.0, 16.8]	11.1 [7.6, 16.0]	12.7 [8.9, 19.5]	< 0.001
Anion gap (mEq/L)	17.0 [14.0, 21.0]	16.0 [14.0, 20.0]	19.0 [16.0, 23.0]	< 0.001
Bicarbonate (mmol/L)	23.0 [20.0, 26.0]	24.0 [21.0, 26.0]	22.5 [19.0, 25.0]	< 0.001
BUN (mg/dL)	33.0 [20.0, 55.0]	30.0 [19. 0, 49.8]	41.5 [24.8, 64.3]	< 0.001
Serum calcium (mg/dL)	8.60 [8.10, 9.20]	8.55 [8.00, 9.10]	8.80 [8.10, 9.30]	0.036
Serum chloride (mEq/l)	106 [101, 111]	106 [101, 111]	104 [99, 110]	0.007
Serum creatinine (mg/dL)	1.60 [1.00, 2.70]	1.40 [0.90, 2.40]	2.00 [1.27, 3.60]	< 0.001
Serum glucose (mg/dL)	146 [117, 197]	148 [119, 200]	144 [111, 191]	0.099
Serum sodium (mEq/L)	139 [135, 142]	139 [135, 142]	138 [134, 143]	0.179
Serum potassium (mEq/L)	4.50 [4.00, 5.10]	4.40 [4.00, 5.00]	4.60 [4.00, 5.30]	0.094
INR	1.80 [1.50, 2.40]	1.70 [1.40, 2.20]	2.20 [1.70, 3.00]	< 0.001
PT (s)	19.7 [16.2, 25.6]	18.8 [15.5, 23.4]	23.0 [18.6, 31.3]	< 0.001
PTT (s)	45.6 [35.3, 63.4]	42.7 [34.1, 59.9]	52.4 [39.5, 70.3]	< 0.001
Albumin (g/dL)	3.00 [2.60, 3.60]	3.00 [2.60, 3.60]	3.10 [2.60, 3.50]	0.710
Bilirubin (mg/dL)	4.20 [1.70, 8.07]	4.20 [1.70, 8.40]	4.10 [1.78, 7.53]	0.433
Urine output (mL)	997 [538, 1534]	1125 [685, 1619]	645 [284, 1221]	< 0.001
RRT	102 (11.5)	65 (10.5)	37 (14.0)	0.160
Vasopressors use	64 (7.2)	28 (4.5)	36 (13.6)	< 0.001
Mechanical ventilation	739 (83.4)	517 (83.1)	222 (84.1)	0.797
SOFA score	10 [7, 13]	9 [6, 12]	12 [9, 15]	< 0.001
SAPS II	44 [34, 54]	41 [33.00, 50]	51 [42, 59]	< 0.001

*Abbreviations* Aids: acquired immune deficiency syndrome, MAP: mean arterial pressure, SpO_2_: oxygen saturation, WBC: white blood cell, BUN: blood urea nitrogen, INR: international normalized ratio, PT: prothrombin time, PTT: partial thromboplastin time, RRT: renal replacement therapy, SOFA: sequential organ failure assessment, SAPS II: simplified acute physiology score II

### Predictors for the death of cirrhotic patients with AKI

The univariate logistic regression analysis showed that cerebrovascular disease, sepsis, heart rate, MAP, respiratory rate, body temperature, SpO_2_, hemoglobin, platelets, WBC, anion gap, bicarbonate, BUN serum chloride, Scr, INR, PT, PTT, urine output, vasopressors use, SOFA score and SAPS II were associated with death in cirrhotic patients with AKI. To obtain the best predictors of mortality in patients with cirrhosis and AKI, we employed a multivariate logistic regression in which backward stepwise regression was used. The mean VIF of 1.58 for the multivariate logistic model indicates the direct absence of multicollinearity among variables. According to the OR in multivariate logistic regression, the high-risk factors of death were male (OR 1.728, CI 1.195-2.500; *p* < 0.05), age (OR 1.026, CI 1.010–1.043; *p* < 0.05), cerebrovascular disease (OR 5.662, CI 3.082–10.401; *p* < 0.05), heart rate (OR 1.023, CI 1.011–1.035; *p* < 0.05), respiratory rate (OR 1.082, CI 1.037–1.129; *p* < 0.05), BUN (OR 1.009, CI 1.001–1.017; *p* < 0.05), Scr (OR 1.197, CI 1.069–1.286; *p* < 0.05), INR (OR 1.297, CI 1.090–1.542; *p* < 0.05), bilirubin (OR 1.030, CI 1.010–1.049; *p* < 0.05) and SOFA score (OR 1.169, CI 1.113–1.228; *p* < 0.05). The OR for cerebrovascular disease in this study was 5.370, and patients with a history of cerebrovascular disease indicated were 5.370 times more likely to die in the hospital than those without a history of cerebrovascular disease. In addition, male patients were 1.982 times more likely to die in the hospital than female patients. On the other hand, body temperature (OR 0.507, CI 0.363–0.708; *p* < 0.05), SpO_2_ (OR 0.862, CI 0.789–0.942; *p* < 0.05), hemoglobin (OR 0.868, CI 0.788–0.956; *p* < 0.05) and urine output (OR 1.000, CI 0.999-1.000; *p* < 0.05) were protective parameters against patient death (Table 2).

**Table 2:** Logistic regression analysis of predictors for death of cirrhotic patients with acute kidney injury

Variables	Univariate				Multivariate		
	OR	95% CI	*P* value		OR	95% CI	*P* value
Cholestasis or alcoholic	1.144	0.857–1.526	0.362				
Sex, male	1.214	0.896–1.647	0.211		1.728	1.195-2.500	0.004
Age (years)	1.002	0.990–1.015	0.700		1.026	1.010–1.043	0.002
Weight (kg)	1.003	0.996–1.009	0.434				
Myocardial infarction	1.467	0.855–2.515	0.164				
Congestive heart failure	0.988	0.684–1.426	0.947				
Peripheral vascular disease	1.193	0.692–2.058	0.525				
Cerebrovascular disease	2.643	1.610–4.341	< 0.001		5.662	3.082–10.401	< 0.001
Chronic pulmonary disease	0.774	0.556–1.076	0.128				
Rheumatic disease	0.409	0.119–1.408	0.156				
Peptic ulcer disease	0.805	0.454–1.425	0.457				
Diabetes	1.014	0.739–1.390	0.932				
Paraplegia	1.423	0.512–3.957	0.499				
Chronic kidney disease	1.113	0.800-1.549	0.524				
Cancer	1.181	0.817–1.706	0.377				
Aids	0.731	0.265–2.017	0.545				
Sepsis	1.577	1.169–2.126	0.003				
Ascites	1.684	1.259–2.252	< 0.001				
Portal encephalopathy	1.589	1.189–2.125	0.002				
Heart rate (beats/minute)	1.015	1.006–1.024	0.001		1.023	1.011–1.035	< 0.001
MAP (mmHg)	0.970	0.956–0.985	< 0.001				
Respiratory rate (beats/minute)	1.099	1.063–1.136	< 0.001		1.082	1.037–1.129	< 0.001
Body temperature (°C)	0.537	0.409–0.705	< 0.001		0.507	0.363–0.708	< 0.001
SpO_2_ (%)	0.874	0.815–0.938	< 0.001		0.862	0.789–0.942	0.001
Hematocrit (%)	0.979	0.953–1.005	0.115				
Hemoglobin (g/dL)	0.918	0.847–0.994	0.034		0.868	0.788–0.956	0.004
Platelets (K/uL)	0.998	0.996-1.000	0.036				
WBC (K/uL)	1.042	1.023–1.062	< 0.001				
Anion gap (mEq/L)	1.080	1.052–1.108	< 0.001				
Bicarbonate (mmol/L)	0.933	0.902–0.965	< 0.001				
BUN (mg/dL)	1.013	1.008–1.018	< 0.001		1.009	1.001–1.017	0.031
Serum calcium (mg/dL)	1.022	0.896–1.166	0.747				
Serum chloride (mEq/l)	0.977	0.958–0.996	0.020				
Serum creatinine (mg/dL)	1.147	1.058–1.243	0.001		1.197	1.069–1.286	0.003
Serum glucose (mg/dL)	0.999	0.997-1.000	0.137				
Serum sodium (mEq/L)	0.989	0.966–1.013	0.357				
Serum potassium (mEq/L)	1.149	0.991–1.333	0.067				
INR	1.680	1.453–1.943	< 0.001		1.297	1.090–1.542	0.003
PT (s)	1.054	1.039–1.070	< 0.001				
PTT (s)	1.009	1.004–1.013	< 0.001				
Albumin (g/dL)	0.905	0.716–1.143	0.403				
Bilirubin (mg/dL)	1.042	1.027–1.058	< 0.001		1.030	1.010–1.049	0.003
Urine output (mL)	0.999	0.999-1.000	< 0.001		1.000	0.999-1.000	< 0.001
RRT	1.397	0.907–2.152	0.130				
Vasopressors use	3.350	1.998–5.617	< 0.001				
Mechanical ventilation	1.074	0.726–1.587	0.722				
SOFA score	1.212	1.164–1.261	< 0.001		1.169	1.113–1.228	< 0.001
SAPS II	1.051	1.039–1.064	< 0.001				

*Abbreviations* OR: order ratio, CI: confidence interval, Aids: acquired immune deficiency syndrome, MAP: mean arterial pressure, SpO_2_: oxygen saturation, WBC: white blood cell, BUN: blood urea nitrogen, INR: international normalized ratio, PT: prothrombin time, PTT: partial thromboplastin time, RRT: renal replacement therapy, SOFA: sequential organ failure assessment, SAPS II: simplified acute physiology score II

### Nomogram construction and validation

**Fig. 2:**
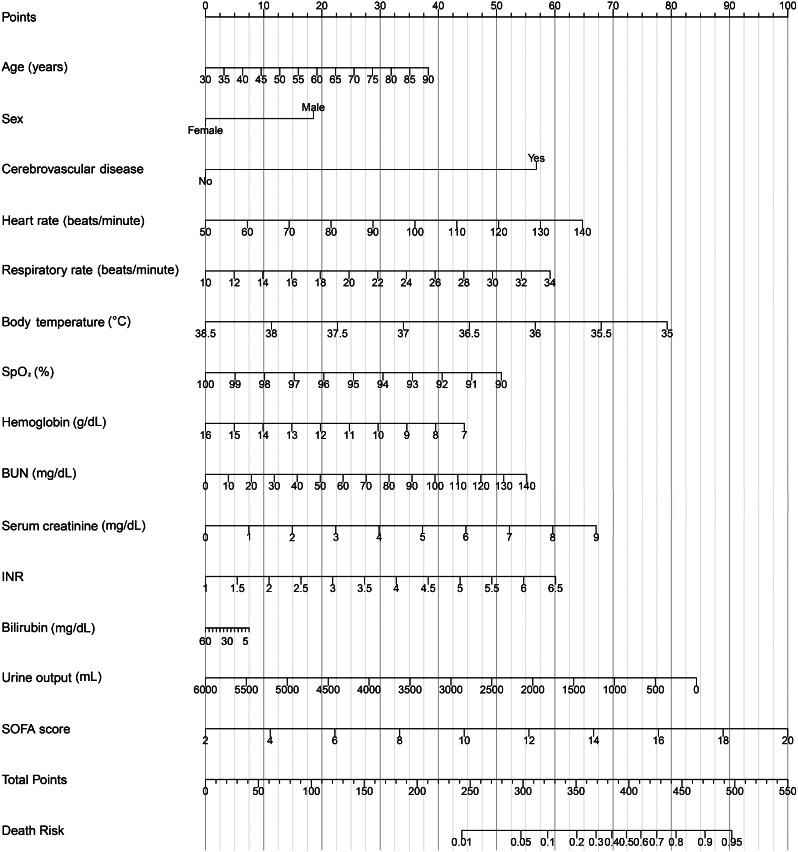
Nomogram for predicting in-hospital death of cirrhotic patients with acute kidney injury. *Abbreviations* INR: international normalized ratio, SOFA: sequential organ failure assessment

A prognostic nomogram for early recognition of in-hospital mortality in cirrhotic patients with AKI was constructed using the multivariate logistic regression results, and points were assigned to the identified factors according to their regression coefficients (Fig. 2). As shown in the nomogram, patients with an elderly age; male; a history of cerebrovascular disease; higher heart rate, respiratory rate, BUN, Scr, INR and SOFA score were more likely to die. Nevertheless, patients with higher body temperature, SpO_2_, hemoglobin, and urine output had a lower risk of death. In addition, the nomogram illustrated higher SOFA score or lower urine output as the most significant contributors to death.

**Fig. 3:**
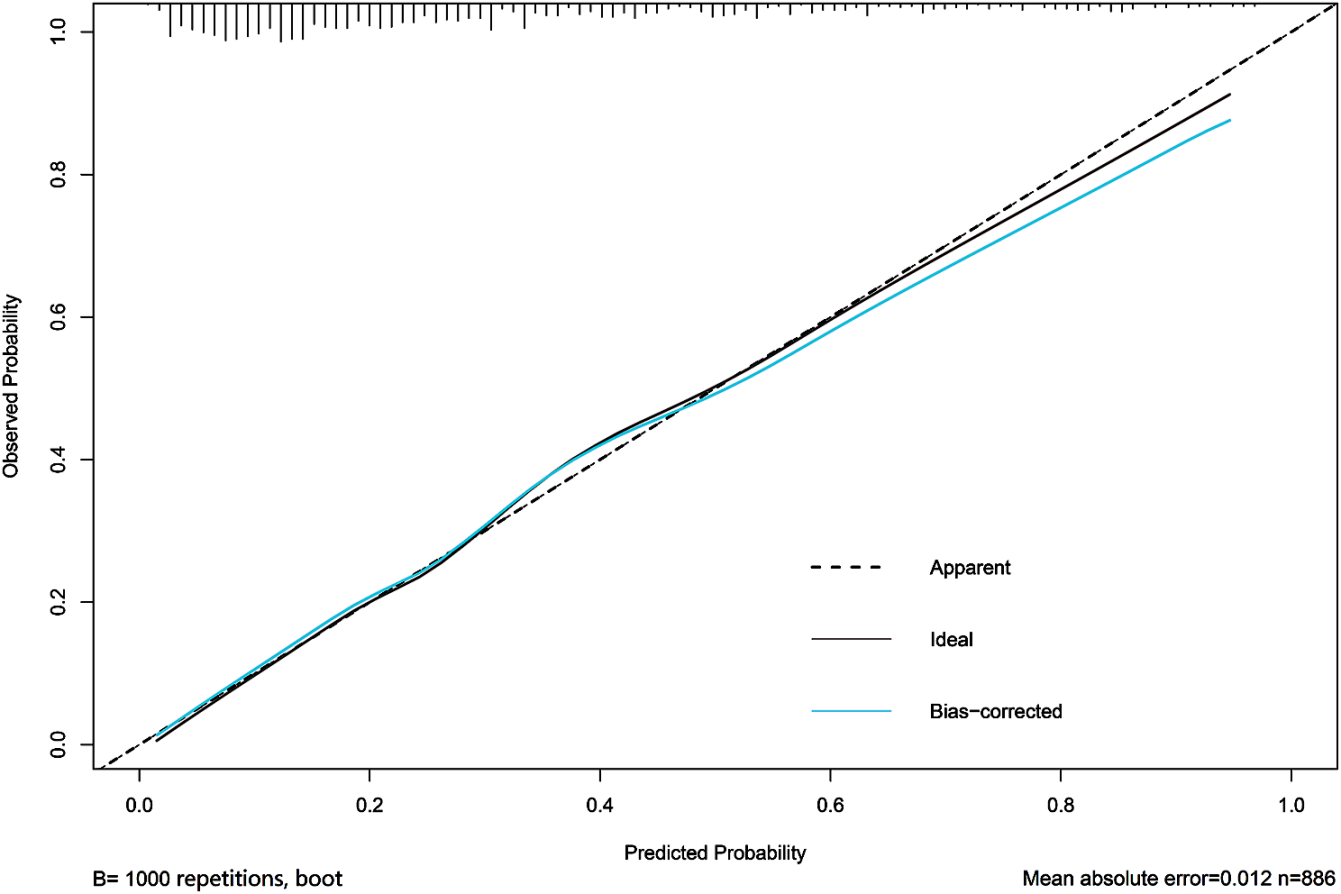
Calibration plots of internal validation

**Fig. 4:**
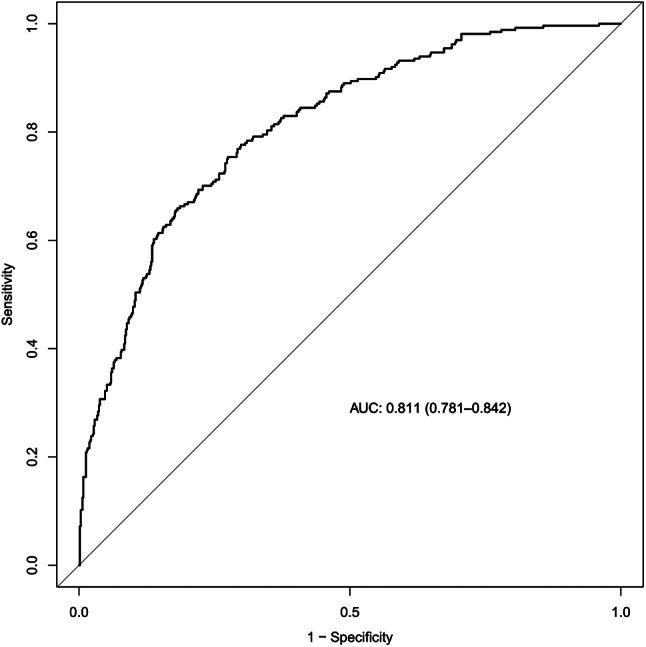
Receiver operating characteristic curve of nomogram. *Abbreviations* AUC: area under curve

**Fig. 5:**
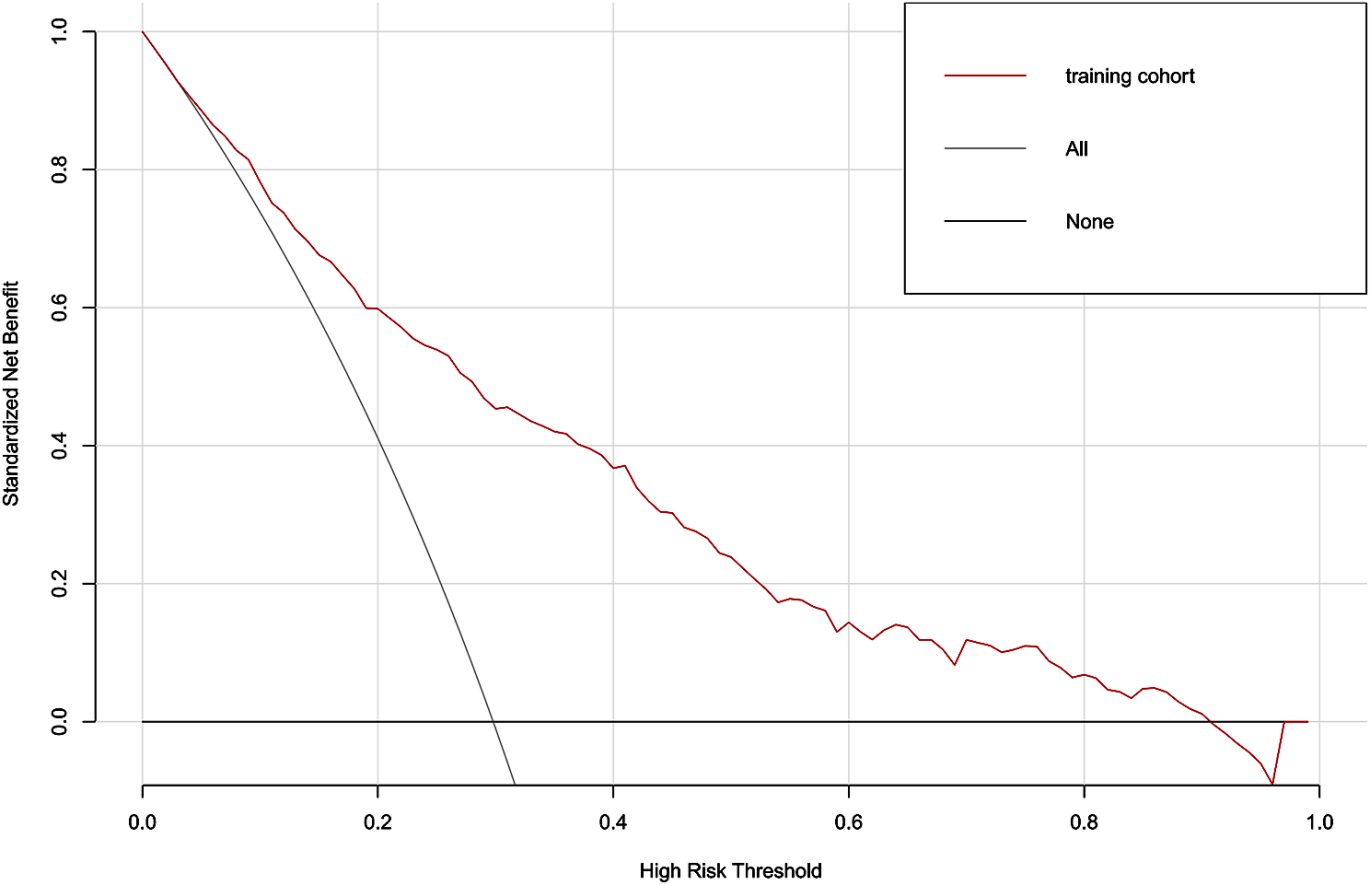
Decision curve analysis curve of the nomogram

A bootstrapping technique with 1000 resamples as qualified by C-Index was used for internal validation to assess the model’s discriminatory power and reduce overfitting bias. In our investigation, the C-Index for the primary cohort was 0.811, the C-Index for the internal validation cohort was 0.796, and the calibration plot demonstrated excellent agreement between the nomogram prediction and the actual observations of mortality (Fig. 3). The AUC of the receiver operating characteristic curve was 0.811, 95% CI: 0.781–0.842, which indicates that the model has good predictive accuracy (Fig. 4). In addition, DCA curve showed that the nomogram model had high clinical value in the range of 1-96%, which further demonstrated that the nomogram model could accurately predict in-hospital mortality in cirrhotic patients with AKI (Fig. 5).

## Discussion

Cirrhosis is caused by numerous acute and chronic liver disorders. AKI is common and deadly in cirrhotic individuals, contributing to an unacceptable mortality rate [15]. The research also shows that the mortality rate of AKI is significantly higher in cirrhotic patients admitted to ICU than in those admitted to ordinary wards [16, 17]. Despite discovering numerous treatment options, the prognosis for AKI in patients with cirrhosis remains poor. Therefore, it is critical to construct an effective model to identify cirrhotic patients with AKI at higher risk of in-hospital mortality, which may help guide treatment. In this investigation, we used logistic regression analysis to identify risk variables for in-hospital mortality in cirrhotic patients with AKI, including age, gender, cerebrovascular disease, heart rate, respiration rate, temperature, SpO_2_, hemoglobin, BUN, Scr, INR, bilirubin, urine volume, and the SOFA score. These risk factors were combined into a nomogram, which showed superior discrimination in predicting patient mortality.

Despite the abundance of research into the causes of AKI in cirrhotic patients, only a few studies have attempted to predict mortality in patients with cirrhosis and AKI [9, 18]. The nomogram illustrated that the SOFA score, urine output, and body temperature were the three variables with the most predictive power for death in patients with cirrhosis and AKI. The SOFA score measures the burden of organ dysfunction to quantify organ impairment. Researchers discovered a strong link between the SOFA score and clinical outcomes, with higher scores often indicating a poor prognosis [19]. The results of this study show that the SOFA score is an independent predictor of mortality in patients with cirrhosis and AKI. In patients with cirrhosis and AKI, urine volume was also a risk factor for death. Oliguria is prevalent in intensive care unit patients and is the underlying cause of renal parenchymal damage [20]. Several studies have demonstrated a correlation between lower urine output and poor outcomes in critically ill individuals [21]. Temperature is a frequently assessed vital indicator for all ICU-admitted patients. Inconsistent reports exist about the effect of fever on mortality in intensive care unit (ICU) patients; some research implies that fever may contribute to mortality, while another meta-analysis suggests that the presence of fever per se may not increase mortality [22]. According to research by Laupland et al., who looked at data from 20,466 adult ICU patients, hypothermia is a significant risk factor for death in the medical ICU [23]. Our study also identified low body temperature as a risk factor for mortality among cirrhotic individuals with AKI. Therefore, hypothermia may be a significant and potentially controllable risk factor for death in patients with cirrhosis and AKI. In addition, age, gender, cerebrovascular disease, heart rate, respiration rate, SpO_2_, hemoglobin, BUN, Scr, INR, and bilirubin were also identified as possible risk factors for death in patients with cirrhosis and AKI.

Based on these probable risk factors of death, a quantitative and graphical nomogram was built for the purpose of predicting the in-hospital mortality of cirrhotic patients who were diagnosed with AKI in the current investigation. The nomogram could determine the scores relating to each potential risk factor. Nomogram, a single numerical estimate of the probability of an occurrence, has demonstrated superior performance than other methods of determining the chance of treatment success, complications, and mortality [24]. To our knowledge, our research is the first to develop a nomogram for predicting hospital mortality in cirrhotic patients with AKI, which can aid in the early identification and care of individuals at high risk.

In our study, we aimed to develop and validate a nomogram for the prediction of in-hospital mortality in patients with cirrhosis with AKI. Wan et al. evaluated overall survival in patients with cirrhosis with AKI using data from the MIMIC IV database, which provided valuable insights into long term prognostic factors [9]. Liao et al. developed nomograms to predict 15 and 30 day survival based on admission data from cirrhotic patients with AKI in the same dataset [25]. Although their study shares some similarities with ours, such as the use of MIMIC IV data and the focus on AKI in cirrhotic patients, there are several important differences. First, our nomogram was specifically designed to predict in-hospital mortality, enhancing the clinical utility of its immediate risk assessment. Second, we used rigorous methods, including multivariate logistic regression analysis and validation techniques, to develop and validate the nomogram. In addition, our study identified and incorporated other predictors not addressed in the studies by Wan et al. and Liao et al. such as cerebrovascular disease, body temperature, and SpO_2_, thereby improving the predictive accuracy and clinical applicability of the nomogram. In addition, Feng et al. developed a nomogram aimed at predicting AKI in cirrhotic patients upon ICU admission [26]. Their study provides valuable insights into early identification and risk stratification of AKI in this patient population, which is crucial for guiding clinical decision-making and interventions during the critical phase of ICU admission. In comparison to our study, which focuses on predicting in-hospital mortality in cirrhotic patients with AKI, Feng et al.‘s study addresses a different aspect of AKI management by targeting its early detection and prediction upon ICU admission. Our study complements the work of Feng et al. by providing a predictive model specifically tailored for predicting in-hospital mortality, which assists in identifying patients at imminent risk of death and guiding timely interventions and intensive monitoring during the hospital stay.

This study has some significant limitations. First, this was a retrospective modeling study conducted at a single location utilizing the MIMIC IV database, and we could not determine the causal relationship between features and outcomes. To confirm the efficacy of our method, we require additional prospective randomized clinical trials. Second, our study design’s retrospective and observational nature may lead to selection bias. Thirdly, we calculated specific missing data using padding, which may have resulted in disparities between the estimated and actual figures. Fourthly, even though our study employed bootstrapping technology for internal validation, future research will also require external validation. Fifthly, our study may not have captured all therapeutic measures that could influence outcomes in this patient population. The complexity of patient management in cirrhotic patients with AKI involves various therapeutic interventions, including medical treatments, invasive procedures, and supportive care measures, which may have an impact on patient outcomes. The absence of comprehensive data on all therapeutic interventions represents a limitation of our study and may have influenced the interpretation of the elaborated data. Finally, our study focused on the prognosis of cirrhotic patients with AKI in the ICU, and the findings may not apply to cirrhotic patients with AKI in non-ICUs and to AKI patients without cirrhosis in ICUs.

## Conclusions

In conclusion, age, sex, cerebrovascular illness, heart rate, respiration rate, body temperature, SpO_2_, hemoglobin, BUN, Scr, INR, bilirubin, urine output, and SOFA score were possible mortality risk factors. In addition, a validated nomogram model based on possible risk variables was successfully developed to predict the in-hospital death of cirrhotic patients with AKI. The nomogram model may improve the identification of in-hospital deaths in patients with cirrhosis combined with AKI and contribute to timely intervention to improve patient prognosis.

## Data Availability

The datasets presented in the current study are available in the MIMIC-IV database (https://physionet.org/content/mimiciv/1.0/).
